# Genomic Resources of *Magnaporthe oryzae *(GROMO): A comprehensive and integrated database on rice blast fungus

**DOI:** 10.1186/1471-2164-10-316

**Published:** 2009-07-15

**Authors:** Shalabh Thakur, Sanjay Jha, Subhankar Roy-Barman, Bharat Chattoo

**Affiliations:** 1 Centre for Genome Research, Department of Microbiology and Biotechnology Centre, Faculty of Science, The M. S. University of Baroda, Vadodara – 390002, India

## Abstract

**Background:**

*Magnaporthe oryzae*, rice blast fungus, is the most devastating pathogen of rice. It has emerged as a model phytopathogen for the study of host-pathogen interactions. A large body of data has been generated on different aspects of biology of this fungus and on host-pathogen interactions. However, most of the data is scattered and is not available as a single resource for researchers in this field.

**Description:**

Genomic Resources of *Magnaporthe oyzae *(GROMO), is a specialized, and comprehensive database for rice blast fungus, integrating information from several resources. GROMO contains information on genomic sequence, mutants available, gene expression, localization of proteins obtained from a variety of repositories, as primary data. In addition, prediction of domains, pathways, protein-protein interactions, sumolyation sites and biochemical properties that were obtained after computational analysis of protein sequences have also been included as derived data. This database has an intuitive user interface that shall prompt the user to explore various possible information resources available on a given gene or a protein, from a single source.

**Conclusion:**

Currently, information on *M. oryzae *is available from different resources like BROAD MIT *Magnaporthe *database, *Agrobacterium tumefaciens*-mediated transformation (ATMT) *M. oryzae *database, *Magnaporthe grisea – Oryza sativa *(MGOS) and Massive Parallel Signature Sequencing (MPSS) databases. In the GROMO project, an effort has been made to integrate information from all these databases, derive some new data based on the available information analyzed by relevant programs and make more insightful predictions to better understand the biology of *M. oryzae*. The database is currently available at:

## Background

*M. oryzae*, previously called *M. grisea*, is the most devastating fungal pathogen of rice, accounting for more than 10 million tons of yield loss every year [1]. This fungus is a haploid filamentous ascomycete (class Pyrenomycetes). Members of *M. oryzae *species complex are also reported to cause disease on many other economically important crops such as barley, wheat, and millet [2]. The rice blast fungus invades rice plants in a manner typical of many foliar pathogens by producing specialized infection structures called 'appressoria'. In nature, rice blast fungus attacks all above-ground parts of rice plants, and seedlings can be killed during epidemics. Under laboratory conditions, root infection of wheat and rice seedlings by *M. oryzae *has also been reported [3,4].

Rice blast pathosystem has emerged as a model to study plant-pathogen interactions because genome sequence information on both the host [5] as well as the pathogen [6] is available and they are both amenable to genetic manipulation. *M. oryzae *was the first plant fungal pathogen to be sequenced. Analysis of *M. oryzae *genome is providing valuable insights on fungal pathogenesis [1].

*M. oryzae *genome is predicted to contain 12,841 [7] and 11,074 (as per BROAD MIT database; version 6) genes [8], but the function of more than 70% of genes is still unknown. A few avirulence/pathogenicity genes have been characterized recently [9]. One of the challenges in post-genomic era is to identify all transcribed regions and experimentally assign gene functions to *M. oryzae *genome. Although computational programs have played an important role in genome annotation, experimental evidence is needed to validate predicted functions. Availability of complete genome sequence of *M. oryzae *provides an opportunity to design insightful experiments so as to understand molecular mechanisms of pathogenesis. Expression of various genes has been analyzed in mycelium and appressorium using MPSS, robust-long serial analysis of gene expression (RL-SAGE) and oligoarray methods [10].

We have developed a comprehensive database, GROMO, integrating available information on genome, transcriptome, and proteome, thus, providing a useful resource for studies on functional genomics in the rice blast fungus. Data on this fungus were obtained from various resources like National Center for Biotechnology Information (NCBI) [11], BROAD MIT [8], MGOS [12], Kyoto Encyclopedia of Genes and Genomes (KEGG) [13], DNA Data Bank of Japan (DDBJ) [14], *M. oryzae *ATMT database [15], etc. Programs written using Perl language were used to analyze the data obtained from various resources and extract necessary information. The programs were also used to establish a connection with various databases for accessing the data using web services provided by the databases. All extracted information from the analyzed data was compiled in a back-end database created using MySQL for construction of GROMO. Some of the information incorporated in GROMO was also generated by correlating the extracted information from different resources with one another. Thus, a comprehensive resource on *M. oryzae *was built on protein domains, pathways, protein localization, presence of sumoylation sites, expression data, protein interacting partners, availability of mutants, etc. This database not only provides researchers an opportunity to extract detailed biological information on a specific gene or protein from a single resource but also prompts the researcher to explore new territories in fungal genomics.

## Construction and content

### Primary Data

#### Sequence Data

Sequence information on *M. oryzae *proteins as available on BROAD, MIT database [11] was downloaded. Sequences from this database have unique locus tags which were used during the analysis for distinguishing sequences from each other. A large number of sequences obtained were described as either hypothetical or predicted. Naming convention for the sequences has been described at BROAD, MIT website.

#### Mutation Data

Information on various *M. oryzae *mutants was obtained from MGOS database and ATMT database for *M. oryzae *which contains information on 21,070 hygromycin-resistant *M. oryzae *(strain KJ201) mutants generated through a large-scale insertional mutagenesis using ATMT [15]. Information on 321 and 299 locus tags and their phenotypes was available for mutants from ATMT and MGOS respectively.

#### Expression Data

MGOS is a web-based database that contains expression profile data on rice-blast fungus interactions. It provides information on gene expression for both rice as well as *M. oryzae *during compatible interaction. Expression profiles obtained using techniques like Expressed Sequence Tags (ESTs) and Serial Analysis of Gene Expression (SAGE) on *M. oryzae *were downloaded and incorporated in our MySQL database file as a part of the back-end database. MPSS expression profile data on *M. oryzae *genes was obtained from MPSS database [9]. Total number of genes for which EST contigs were found was 2,629. MPSS tags of 17-bp and 20-bp signatures were found in 3,911 genes and 5,095 genes were found to have SAGE tags.

#### Localization Data

**Figure 1:**
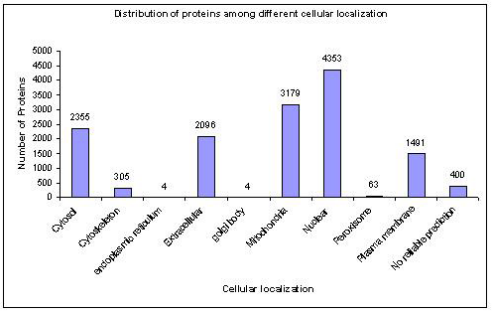
**Distribution of localization of various proteins in *M. oryzae***.

Information on localization of *M. oryzae *proteins was obtained from the e-Fungi database [16] for version 5 sequences and was analyzed using WoLF-PSort and SignalP program for version 6 sequences. The e-Fungi database provides fungal biologists with a resource for comparative studies of a large range of fungal genomes. Its analysis library supports comparative study of genomic data, functional annotation, and results of large scale analyses of all genomes in the database. Localization of *M. oryzae *proteins in e-Fungi database were determined using PSort [17], WoLF-PSort [18] and SignalP [19] programs. PSort is a program that predicts subcellular localization of proteins by exploiting comprehensive knowledge of protein sorting. WoLF-PSORT predicts subcellular localization sites of proteins based on their amino acid sequences. This method, which is a major extension to the PSORTII program, makes predictions based on both known sorting signal motifs and some correlative sequence features such as amino acid content. SignalP 3.0 server predicts the presence and location of signal peptide cleavage sites in amino acid sequences from different organisms. The method incorporates a prediction of cleavage sites and a signal peptide/non-signal peptide prediction based on a combination of several artificial neural networks and Hidden Markov Models. Distribution of cellular localization for various proteins in *M. oryzae *is shown in Figure 1.

### Derived Data

#### Domains

Domain analysis of proteins was performed on *M. oryzae *protein sequences using SMART [20] and Pfam [21] databases. SMART allows identification and annotation of genetically mobile domains and domain architecture. More than 400 domain families found in signaling, extracellular and chromatin-associated proteins are extensively annotated with respect to phyletic distribution, functional class, tertiary structures and functionally important residues. Pfam is a large collection of multiple sequence alignments and Hidden Markov Models covering many common protein domains and families. This database has two parts; first one is the curated part of Pfam containing over 9,318 protein families and second is a supplement called Pfam-B which contains a large number of small families taken from PRODOM database [22] that do not overlap with Pfam-A. All protein sequences from *M. oryzae *were scanned through using the web services of SMART database with an E-value cut-off of 1. A total of 1,878 domains were found for 7,674 protein sequences from SMART database. Whereas, 2,573 domains were predicted in *M. oryzae *for 5,986 proteins using hmmpfam from Pfam database. Final analysis showed 3,220 domains predicted for 9,815 proteins and were incorporated in GROMO as shown in Table 1.

**Table 1:** Results of domain analysis performed using SMART and Pfam

	SMART	Pfam	Unique Smart	Unique Pfam	Common	Total
Domains	1878	2573	647	695	1878	3220
No. of Genes	7674	5986	3829	2141	3845	9815

#### Pathways

Putative pathways were predicted for *M. oryzae *protein sequences using KEGG Pathway database. KEGG PATHWAY is a collection of manually drawn pathway maps representing information on the molecular interaction and metabolic networks. In KEGG, 74,729 pathways are generated from 361 reference pathways. KEGG Application Programming Interface (API) was used for retrieving information from the database for analysis of putative pathways in *M. oryzae. M. oryzae *proteins were compared to Swiss-Prot database [23,24] using BlastP [25] with an E-value cut-off of 10^-3^. Top 30 hits from blast result of each protein sequence were selected and checked in KEGG for prediction of biological pathways in which they were involved. Each query protein sequence from *M. oryzae *was assigned probable pathways on the basis of pathways obtained from KEGG database for their homologous protein sequences in other species (See Additional file 1: Schematic representation of pathway prediction in *M. oryzae***)**. Approximately 219 different pathways were predicted for 2,477 proteins in *M. oryzae*.

#### Protein-Protein Interactions

Probable protein-protein interactions in *M. oryzae *were predicted using interactome information for *Saccharomyces cerevisiae *from Center of Cancer Systems Biology (CCSB) Yeast Interactome Database at Dana-Farber Cancer Institute and Harvard Medical School [26]. Interaction data from the database was obtained as downloadable tab-delimited files. Protein interactions in *M. oryzae *were predicted on the basis of ortholog interaction data in *S. cerevisiae*. KEGG database was scanned to find orthologs of *M. oryzae *in *S. cerevisiae *and vice versa with an E-value cut off of 10^-3^. A total of 2,083 *M. oryzae *orthologs in *S. cerevisiae *that resulted from this study were subjected to scan against interactome data from CCSB Yeast Interactome database for prediction on protein-protein interactions (See Additional file 2: Schematic representation showing prediction of protein-protein interactions in *M. oryzae*).

#### Sumoylation sites

Putative sumoylation sites in *M. oryzae *proteins were predicted using SUMOsp 2.0 software for sumoylation site prediction by Cuckoo workgroup [27]. The non-redundant training data in software contained 279 sumoylation sites from 166 distinct proteins. SUMOsp 2.0 predicted 6,000 sumoylation sites for 4,494 protein sequences in *M. oryzae *at a high cut-off value.

#### Biochemical properties

Biochemical properties of the protein sequences were calculated using Pepstats program [28] from European Molecular Biology Open Software Suite (EMBOSS) package [29]. EMBOSS is a free Open Source software analysis package specially developed for the needs of the molecular biology user community. Pepstats was programmatically linked and used to predict biochemical properties of *M. oryzae *proteins. Pepstats calculated molecular weight, isoelectric point, charge, size of protein, extinction coefficient and average residue weight for all the proteins.

The GROMO database is currently available at: 

### Architecture and Design of GROMO

The architecture and design of GROMO (See Additional file 3: Overview of GROMO architecture and Design) consists of four tiers. T1: User Interface developed using HTML, T2: Apache web server [30] and programs for sequence analysis and information retrieval, T3: MySQL database [31] storing analysis data and T4: Perl CGI scripts [32] for retrieving and displaying analysis results for selected gene(s). All scripts and program in T2 and T4 are accessible from the analysis information page generated for each gene.

### Tier 1: User Interface

User interface provides the user access to GROMO using various input queries and provide links to additional information pages which guide the user during browsing of GROMO. The query inputs from user interface are sent to programs in layer T2 via post method.

### Tier 2: Programs for sequence analysis and information retrieval

Apache web server receives query request from user interface and sends it to Perl CGI script in T2 for retrieving locus tag and gene description information from MySQL database. Program in T2 also use BLAST program obtained from NCBI ftp site for sequence based analysis and parse BLAST result to represent necessary information on browser.

### Tier 3: Database Schema

The Relational Database Management System MySQL [32] was used to store data integrated in GROMO. DBD::mysql (Database Driver) and DBI (Database Interface) module are used in Perl CGI scripts for accessing data from MySQL database. Database schema was divided into different tables depending on the type of data incorporated. The description of different tables of schema is given in the additional file 4: Description of information contained in different tables of relational database.

### Tier 4: Scripts retrieving and displaying analysis result for selected gene

Perl CGI scripts in this layer retrieve analyzed information from MySQL database for selected locus tag from result page and display it on browser. "Export" option for downloading analyzed information in the form of text file is provided on summary section for selected gene.

## Utility

### Web Interface Access

Data stored in GROMO can be accessed through web interfaces which have been generated using HTML. It gives entry point to explore the data stored in GROMO by following two different kinds of links provided: (i) Search using Keyword (ii) Link to BLAST search.

### Search using Keyword

**Figure 2:**
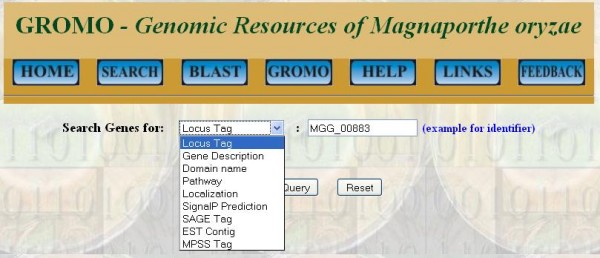
**Screenshot of web interface showing various input options accepted by GROMO**.

**Figure 3:**
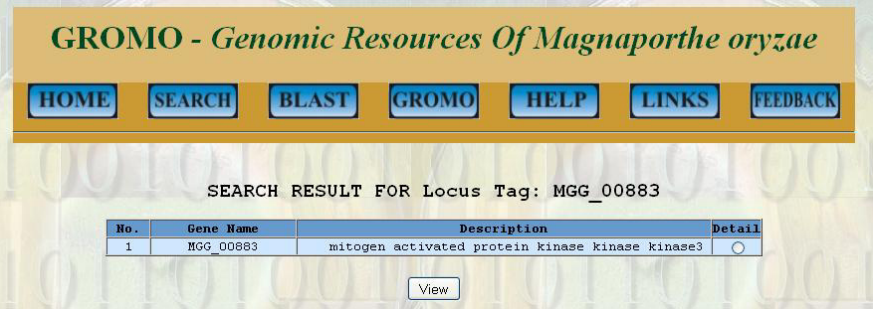
**Screenshot of the query result page showing gene with specific locus tag and its description**.

This feature of GROMO allows the user to browse database by inputting keyword for selected query option. There are nine query options (Figure 2) which accept specific keyword input and corresponding data can be retrieved from the database. Description of each query option and their keyword input is given in "Help" section of GROMO. Information in "Help" section includes information about query options, examples of keyword inputs, etc. Output formats for all query options remain the same; it includes list of protein IDs and their descriptions which satisfy the search criteria and are retrieved from the database (Figure 3).

### BLAST Search

BLAST-based search allows user to browse GROMO using sequence in FASTA format. With a query sequence, BLAST generates a table containing best hits in *M. oryzae *which are organized according to percent identity between the sequences.

### Representation of analysis results

**Figure 4:**
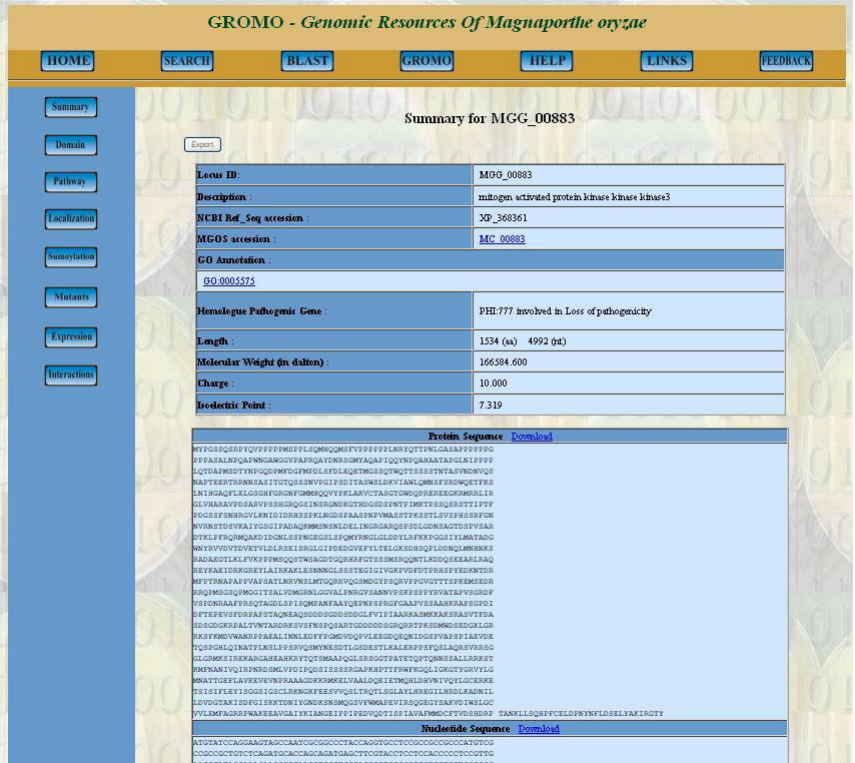
**Screenshot presenting summary section of MGG_00883 along with links for other sections and web pages**. *It provides general information about the gene/protein describing its biochemical properties, accession number, annotations and sequences*.

The results of Keyword Search and BLAST Search display locus tag and description of gene or protein which provide link to summary section (Figure 4) for selected locus tag from where information in different sections can be accessed. Sections such as Domains, Pathways, Localization, Sumoylation, Mutants, Expression and Interactions contain analyzed results for the corresponding gene or protein.

### Other web interfaces

Other web interfaces includes "Help" section which provides information about the inputs accepted and output generated. "Link" section provides links to external web resources and groups working on *M. oryzae*. "Feedback" form provides user with an option to post their comments and queries about the database. "GROMO" section gives overall diagrammatic representation of complete database and its contents. User can submit any valid new information available about genes and proteins of *M. oryzae *using the format given on home page of the database.

## Discussion

The queries provided by GROMO are focused on retrieving details available from various databases along with queried information for particular a gene or protein in *M. oryzae*. Currently, information about this fungus is dispersed in different resources at different locations. Among the existing databases, (i) BROAD MIT provides sequence information on *M. oryzae *genes and proteins with some information on protein domains; (ii) *M. oryzae *ATMT database provides information on some mutants and (iii) Information on gene expression during host-pathogen interactions is available from MGOS and MPSS databases.

In the GROMO project, an effort has been made to bring together information on *M. oryzae *from various resources and develop a comprehensive database which consists of experimental and computational data. GROMO database provides researcher information not only on gene and protein sequences but also on possible domains present in a protein, predicted pathways, probable interacting partners, sub-cellular localization, protein sumoylation sites and even biochemical properties of any protein. In addition to a common blast search, GROMO provides the user the possibility of keyword search using the options like locus tag, domain name, pathway, localization, SignalP prediction, SAGE tag, MPSS tag, EST ID and mutants. Moreover, some of the experimental data obtained from external resources are represented in more interpretable form, thus, providing researchers with a better understanding about the fungus in order to help design critical experiments to gain deep insights into biology of fungi in general and rice blast in particular. In order to incorporate newer findings, the database will be updated every 6 months. The directly submitted data will be cross-checked against all existing databases and will be uploaded to GROMO with all the possible predicted features for a specific protein.

## Conclusion

GROMO is a composite and unique resource database on rice blast fungus. In this project, an effort has been made to integrate information from various public databases, derive some new data based on available information resources and make more intuitive predictions to better understand the biology of this fungus. Many scientists are working actively on functional characterization of genes of the blast fungus, expression profiling during host-pathogen interactions and also in the field of proteomics. Updating the current information on blast functional genomics using newer findings coupled with the use of bioinformatics tools will be an area of future interest. *Magnaporthe *research community is encouraged to submit its research findings periodically so that this unique resource database can be kept up to date.

## Availability and requirements

Project Name: GROMO – Genomic Resources of *Magnaporthe oryzae*

Project homepage: The database is currently available at 

Operating system(s): Platform independent (web server)

Programming language(s): HTML, Perl, CGI

License: Free for academics, Authorization is needed for commercial use

(Please contact the corresponding author for more details)

## Authors' contributions

ST developed programs, scripts, tools for the database, carried out data analysis and helped in drafting the manuscript; SJ helped in conceiving and designing the web server idea and analyzing the data; SRB and BBC wrote the manuscript and provided critical inputs to improve the manuscript. BBC also coordinated the GROMO project. All authors read and approved the final manuscript.

## Supplementary Material

Additional file 1 **Schematic representation of pathway prediction**. Schematic representation of pathway prediction in *M. oryzae *showing various steps performed during the analysis. Steps of analysis are divided into two parts (i) Sequence based analysis using BLAST and SwissProt and (ii) Pathway information searching using KEGG. Click here for file

Additional file 2 **Schematic representation showing prediction of protein-protein interactions**. Schematic representation showing prediction of protein-protein interactions in *M. oryzae *using protein interaction information from CCSB Yeast Interactome database and homology searching using KEGG. Click here for file

Additional file 3 **Overview of gromo architecture and design**. Overview of GROMO architecture and Design showing tiers involved in the construction of database. Solid lines in the figure represents inputs passed through different tiers and dotted lines represent output. Click here for file

Additional file 4 **Description of information contained in different tables of relational database**. Description of information contained in different tables of relational database. Click here for file
