# Supplementary figures and images for: Genomic Resources of Magnaporthe oryzae (GROMO): A comprehensive and integrated database on rice blast fungus
Source: BMC Genomics. 2009 Jul 15;10:316. doi: 10.1186/1471-2164-10-316 (PMC2721851; doi:10.1186/1471-2164-10-316)

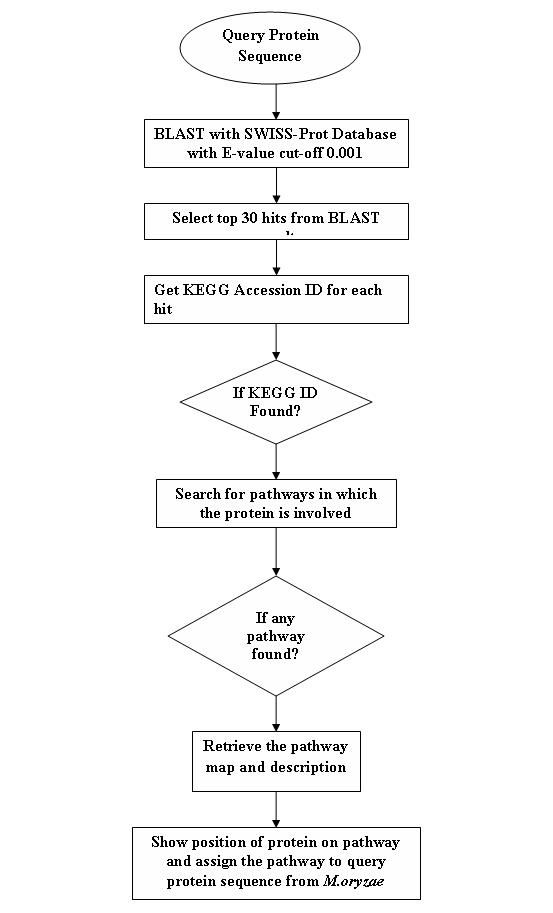

Supplement: Additional file 1 — Schematic representation of pathway prediction. Schematic representation of pathway prediction in M. oryzae showing various steps performed during the analysis. Steps of analysis are divided into two parts (i) Sequence based analysis using BLAST and SwissProt and (ii) Pathway information searching using KEGG. [file 1471-2164-10-316-S1.jpeg]

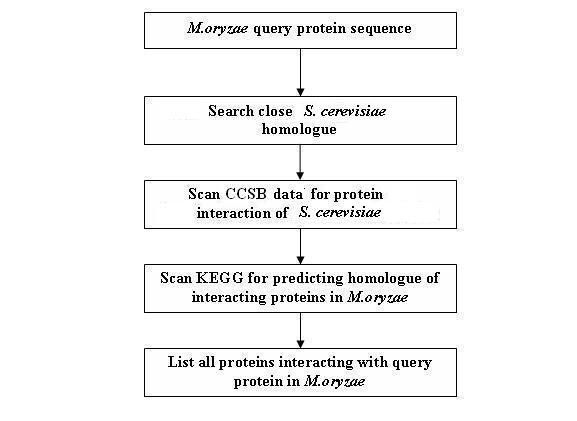

Supplement: Additional file 2 — Schematic representation showing prediction of protein-protein interactions. Schematic representation showing prediction of protein-protein interactions in M. oryzae using protein interaction information from CCSB Yeast Interactome database and homology searching using KEGG. [file 1471-2164-10-316-S2.jpeg]

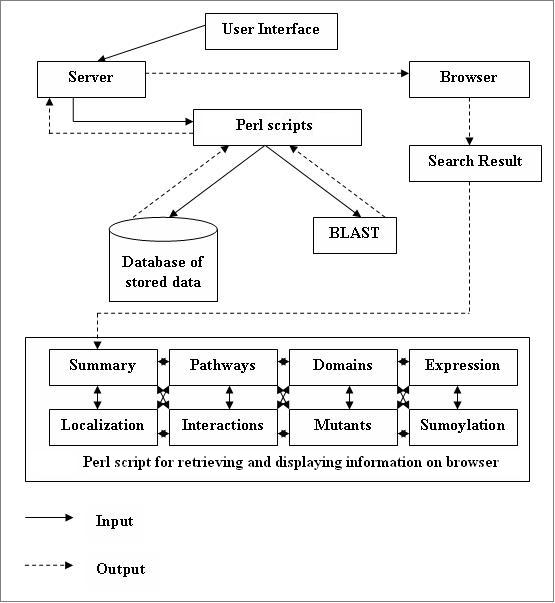

Supplement: Additional file 3 — Overview of gromo architecture and design. Overview of GROMO architecture and Design showing tiers involved in the construction of database. Solid lines in the figure represents inputs passed through different tiers and dotted lines represent output. [file 1471-2164-10-316-S3.jpeg]
