# Supplementary material for: Genomic Resources of Magnaporthe oryzae (GROMO): A comprehensive and integrated database on rice blast fungus
Source: BMC Genomics. 2009 Jul 15;10:316. doi: 10.1186/1471-2164-10-316 (PMC2721851; doi:10.1186/1471-2164-10-316)
Supplement: Additional file 4 — Description of information contained in different tables of relational database. Description of information contained in different tables of relational database. [file 1471-2164-10-316-S4.pdf]

Additional file 4: Description of information contained in different tables of relational database

Table Name	Description
Summary	Contains annotation, sequences and biochemical properties
Domain_details	Contains domain predicted using Pfam and SMART
Pathway_details	Contains predicted pathways using KEGG
Localization_details	Contains predicted localization information
Sumoylation_details	Contains sumoylation sites predicted using SUMOsp 2.0
Mutants_details	Contains mutant data obtained from MGOS and ATMT database
Expression_details	Contains EST, MPSS and SAGE data obtained from MGOS and *Magnaporthe oryzae* MPSS database.
Interaction_details	Contains probable interactions predicted using CCSB database
